# Marine Microbial Fibrinolytic Enzymes: An Overview of Source, Production, Biochemical Properties and Thrombolytic Activity

**DOI:** 10.3390/md20010046

**Published:** 2022-01-02

**Authors:** Noora Barzkar, Saeid Tamadoni Jahromi, Fabio Vianello

**Affiliations:** 1 Department of Marine Biology, Faculty of Marine Science and Technology, University of Hormozgan, Bandar Abbas 74576, Iran; 2 Persian Gulf and Oman Sea Ecology Research Center, Iranian Fisheries Sciences Research Institute, Agricultural Research Education and Extension Organization (AREEO), Bandar Abbas 93165, Iran; 3 Department of Comparative Biomedicine and Food Science, University of Padova, Viale dell’Università 16, 35020 Legnaro, Italy; fabio.vianello@unipd.it

**Keywords:** cardiovascular diseases, fibrinolytic enzymes, marine microorganisms, thrombolytic activity

## Abstract

Cardiovascular diseases (CVDs) have emerged as a major threat to global health resulting in a decrease in life expectancy with respect to humans. Thrombosis is one of the foremost causes of CVDs, and it is characterized by the unwanted formation of fibrin clots. Recently, microbial fibrinolytic enzymes due to their specific features have gained much more attention than conventional thrombolytic agents for the treatment of thrombosis. Marine microorganisms including bacteria and microalgae have the significant ability to produce fibrinolytic enzymes with improved pharmacological properties and lesser side effects and, hence, are considered as prospective candidates for large scale production of these enzymes. There are no studies that have evaluated the fibrinolytic potential of marine fungal-derived enzymes. The current review presents an outline regarding isolation sources, production, features, and thrombolytic potential of fibrinolytic biocatalysts from marine microorganisms identified so far.

## 1. Introduction

Thrombosis is a major cause of cardiovascular diseases (CVDs) including acute myocardial infarction, ischemic heart disease, valvular heart disease, peripheral vascular disease, arrhythmias, high blood pressure and stroke, and it is a leading cause of death worldwide [1]. With population growth, aging and changing lifestyles, thrombotic diseases have become a more serious problem [2]. Thrombin catalyzes the conversion of fibrinogen to fibrin, which is a key component of blood clots or thrombi [3]. Public health data of CVDs is well documented by the World Health Organization (WHO). As for WHO, in 2016 alone, CVDs caused 17.9 million deaths globally [4], with a prediction that approximately 23.3 million people will be affected by 2030 [5]; hence, CVDs are emerging as a global health concern as well as economic burden [2]. Thrombosis is known as one of the foremost causes of CVDs and is characterized by the formation of fibrin clots. During normal physiological conditions, there is a homeostatic balance in the formation and degradation of fibrin; however, in some pathological disorders, it is unbalanced, resulting in aggregation of fibrin resulting in thrombosis [6,7]. The rapid dissolution of blood clots and the re-establishment of blood flow are critical for treating thrombotic diseases effectively.

Fibrinolytic enzymes are deemed as the most promising medication for the clinical treatment of thrombosis [6]. They can be grouped according to their function as plasminogen activators (e.g., tissue-type plasminogen activator t-PA, urokinase plasminogen activator u-PA, streptokinase and plasmin-like (e.g., nattokinase) and lumbrokinase fibrinolytic enzymes [8]. 

Plasminogen activators hydrolyze fibrin by the production of plasmin, and the latter can directly break down fibrin clots [9]. To date, a recombinant tissue-type plasminogen activator (rt-PA) is the only commercial thrombolytic agent with FDA approval. However, clinical data revealed its short time window along with its potential neurotoxicity, and hemorrhages resulted in the possible failure of t-PA treatment [10,11,12] in addition to its short half-life [13,14,15] and low effectiveness [16]. For instance, the high cost and undesirable side effects prompted investigators to explore the cost-effective and safer thrombolytic agents [17]. 

In the last decades, many fibrinolytic enzymes from natural resources, such as snakes [18], earthworms [19,20], insects [21], plants [22], mushrooms [23], microorganisms [24,25] and fermented foods such as Chungkook-jang [26] and Tempeh [27], have been identified and studied. Even though these enzymes have been characterized from a wide range of different sources, microbial fibrinolytic enzymes are considered attractive tools due to their features, such as enhanced specificity [8], low production cost [8], comparatively high yield [28] and the possibility to be genetically modified by recombinant DNA technology and protein engineering approaches [29]. Marine ecosystems serve as a reservoir of microorganisms producing important therapeutic metabolites, especially enzymes [30,31,32,33,34,35,36,37,38,39], but they remain largely unexplored to date. Due to the wide biodiversity of the marine environment, marine microorganisms can provide a diverse array of enzymes for biotechnological development, with possible improved pharmacological properties and lesser side effects [40]. Although many other research studies must be carried out to assess the toxicity of these enzymes, evidence showed minimal side effects upon their application to humans [40,41]. In this view, particular attention should be paid to possible allergenic properties of microbial fibrinolytic enzymes [42]. Hence, fibrinolytic enzymes from marine sources have gathered clinical interest during these decades.

The current review presents an overview regarding the resources, production, properties and thrombolytic activity of fibrinolytic enzymes from marine microbes identified so far.

## 2. Marine Microorganisms as Sources of Fibrinolytic Enzyme

Marine microorganisms are important resources of fibrinolytic enzymes. These enzymes possess potential efficacy for health augmentation and nutraceutical use, and their application could prevent cardiovascular diseases effectively [28]. Marine microorganisms producing fibrinolytic enzymes, including bacteria (*Streptomyces lusitanus* [43], *Streptomyces radiopugnans* VITSD8 [44], *Streptomyces violaceus* VITYGM [45], *Pseudomonas* *aeruginosa* KU1 [46,47], *Alteromonas piscicida* [48], *Pseudoalteromonas* sp. IND11 [49], bacterial strain GPJ3 [50], *Marinobacter aquaeolei* MS2-1 [51], *Bacillus flexus* [52], *Bacillus subtilis* [53], *Bacillus subtilis* HQS-3 [54], *Bacillus vallismortis* [55], *Bacillus subtilis* D21-8 [56], *Bacillus pumilus* BS15 [29], *Bacillus subtilis* WR350 [57], *Bacillus subtilis* JS2 [58], *Bacillus velezensis* BS2 [59], *Bacillus subtilis* ICTF-1 [41], *Shewanella* sp. IND20 [60], *Serratia rubidaea* KUAS001 [61] and *Serratia marcescens* subsp. *sakuensis* [62,63,64], *Arthrospira platensis* [65]) and microalgae (*Chlorella vulgaris* [66,67], *Dunaliella tertiolecta* [68] and *Tetraselmis subcordiformis* [69]), are summarized in Table 1. As shown in Table 1, marine microorganisms that are classified to the genus *Bacillus* are considered as the most valuable resources for the production of fibrinolytic enzymes, while there are no studies that have evaluated the fibrinolytic potential of marine fungal-derived enzymes. It should be noted that the catalytic activity of fibrinolytic enzymes can be improved by chemical modifications and mutant selection [70,71].

## 3. Purification of Fibrinolytic Enzymes

The main purpose of purifying enzymes is to remove other contaminating proteins and other interfering biomolecules. Furthermore, enzyme purification allows the acquisition of insights about structural and functional features of the purified enzyme, as well as foretells its applications [76]. The required level of purity depends on the purpose for which the protein is to be used. If deemed for therapeutic use, the enzyme must have higher level purity and be processed through several subsequent purification steps.

Currently, several approaches have been used for separating and purifying fibrinolytic enzymes from marine microorganisms (Table 2). These approaches involved the extraction of bacteria with aqueous buffer solution as first step, followed by a concentration/precipitation step using acetone or ammonium sulfate and dialysis [41,43,44,52,62,65,66]. As shown in Table 2, the use of ammonium sulfate for protein precipitation is preferred, as it is a low-cost reagent, highly soluble in water and it is able to stabilize proteins and enzymes [41,43,44,47,52,62,65]. Further purification is carried out by employing different chromatographic steps (Table 2). 

Several purification strategies are listed in Table 2, along with their efficiencies (enzyme specific activities obtained). For example, Barros and colleagues (2020) used ammonium sulfate precipitation (40–70%), acetone precipitation, DEAE-Sephadex (anion exchange) and Superdex 75 (size exclusion) chromatography to purify a fibrinolytic enzyme from *Arthrospira platensis*. The eluted enzyme showed a specific activity of 7,988 U/mg with 32.42-fold purification [65]. A fibrinolytic enzyme from *Bacillus subtilis* ICTF-1 was purified by a three-step procedure. As a first step, ammonium sulfate precipitation was adopted for providing suitable protein concentration, followed by UnoQ Sepharose Strong Anion Exchanger and Butyl Sepharose FF chromatography. The enzyme had a molecular mass of 72 kDa [41].

## 4. Biochemical Characterization of Marine Microbial Fibrinolytic Enzymes

### 4.1. Physicochemical Properties of Fibrinolytic Enzymes

#### 4.1.1. Molecular Weight and Effect of pH, Temperature, Inhibitors and Ions

Table 3 provides a detailed overview of the significant physicochemical characteristics of marine microbial fibrinolytic enzymes, including molecular mass, optimal pH and temperature. The molecular mass of the purified marine microbial fibrinolytic enzymes varied significantly, ranging from as low as 21 kDa in an actinomycete (*Streptomyces lusitanus*) [43] to as high as 72 kDa in a cyanobacterium (*Arthrospira platensis*) [65]. Most marine microbial fibrinolytic enzymes have optimum pH fluctuating from neutral to alkaline values, ranging from 6 [65] to 7 [43,44,62] and from 8 [29,52,58,59,60] to 9 [41,75]. The optimal temperature of marine microbial fibrinolytic enzymes ranges between 33 °C (*Streptomyces radiopugnans* VITSD8) [44] to 60 °C (*Bacillus flexus*) [52].

Moreover, some studies have focused on the effect of chemical reagents and metal ions to delineate and characterize catalysis by these novel fibrinolytic enzymes. Table 3 summarizes the effect of metal ions as well as inhibitors on the fibrinolytic enzyme activities. Indeed, according to the specific chemical functionality in their active site, fibrinolytic enzymes can be classified as metalloproteases, serine proteases and serine metalloproteases. As shown in Table 3, the majority of fibrinolytic enzymes from *Bacillus* spp. belongs to serine proteases, and their activity is inhibited by PMSF (Phenyl Methyl Sulphonyl Fluoride). Proteolytic enzymes possessing an active group (OH) from serine amino acid in the catalytic site are recognized as serine proteases. During inhibition of catalytic activity, sulfonyl group of PMSF binds irreversibly to the serine OH group in the active site [77]. In addition, a metalloprotease from *Pseudomonas aeruginosa* KU1 is repressed by some metal ions, e.g., Mn^2+^, Fe^2+^ and Zn^2+^ [47]. Similarly, the activities of fibrinolytic enzymes that belong to serine metalloprotease are dependent on divalent metal ions, such as Mn^2+^, Mg^2+^ and Zn^2+^, for enzymes from *Serratia marcescens* subsp. *sakuensi* [62], Fe^2+^ for enzymes from *Arthrospira platensis* [65] and *Chlorella vulgaris* [66]; thus, their catalyses were inhibited by chelating agents such as EDTA (ethylenediaminetetraacetic acid) and EGTA (ethylene glycol-bis(β-aminoethyl ether)-*N*,*N*,*N*′,*N*′-tetraacetic acid). 

#### 4.1.2. Fibrinogen Lytic Activity

The efficacy of fibrinolytic enzymes is determined by two different mechanisms: indirectly activating the plasminogen and directly acting on fibrins [78]. In Table 4, the activity of different fibrinolytic enzymes isolated from marine microbial sources reported in terms of direct or indirect fibrinogen lytic activity is listed. As shown in Table 4, marine *Bacillus* enzymes are generally directly acting on fibrin forming or fibrin degradation products. AprEBS2 isolated from *Bacillus velezensis* BS2 revealed high Aα fibrinolytic activity, followed by moderate Bβ and mild γ chains fibrinolysis [59]. Nevertheless, fibrinolytic enzymes from *Bacillus pumilus* BS15 [29] and *Bacillus subtilis* JS2 [58] displayed no γ-chain lysis. *Bacillus licheniformis* KJ-31 was one of the microorganisms that only produced fibrinolytic enzymes with high Aα fibrinogen lytic activity [75].

### 4.2. Amidolytic and Kinetic Properties of Marine Microbial Fibrinolytic Enzymes

Microbial fibrinolytic enzymes display amidolytic (or pro-coagulant) activity, which is assessed using different synthetic chromogenic substrates (Table 5). Most of the studied enzymes show high specificity towards N-Succ-Ala-Ala-Pro-Phe-pNA, classifying them as serine proteases [29,41,58,59,75]. In addition, kinetic parameters including the Michaelis constant [79], rate of reaction (V_max_) and the turnover number (k_cat_) help understand the specificity and affinity of an enzyme for a particular substrate [6]. Table 6 summarizes the kinetic properties of selected fibrinolytic enzymes isolated from marine microorganisms in different reaction conditions by using both natural and synthetic substrates.

## 5. Production of Marine Microbial Fibrinolytic Enzymes

### 5.1. Construction of Genetically Engineered Strains

Gene cloning, mutagenesis and recombinant DNA technology have also been employed for the overexpression of fibrinolytic enzymes in bacterial hosts and to engineer their catalytic properties. For example, Yao and colleagues (2018) achieved significantly higher fibrinolytic activity of the recombinant fibrinolytic enzyme from *Bacillus pumilus* BS15 [29]. In another example, Che and colleagues (2020), using gene dosage, codon optimization and process optimization, achieved high expression and secretion of a fibrinolytic enzyme (fibase) isolated from marine *Bacillus subtilis* [71]. Hence, a combination of culture media optimization and recombinant DNA technology has been effectively employed for augmenting the enzyme titer. Table 6 indicates some of the heterologously expressed fibrinolytic enzymes from marine microorganisms.

### 5.2. Fermentation Approach

The production cost of an enzyme is one of the challenging factors for the industrial sector. The commercial obtainability of microbial fibrinolytic enzymes needs high yield at the lowest possible costs [80]. Hence, fermentation approaches are highly remarkable in cutting down the cost of production for an enzyme. For instance, selected submerged fermentations can improve production yield and efficiency. Similarly, Anusree and colleagues (2020), by using submerged fermentation, were able to improve the expression of fibrinolytic enzyme from a bacterium *Serratia rubidaea* KUAS001 obtained from marine milieus [61]. In addition, Pan and colleagues (2019) showed the utilization of non-sterile submerged fermentation to minimize the production cost of enzymes from *Bacillus subtilis* D21-8 [56]. Moreover, several researchers showed that the use and application of diverse statistical tools, such as Box–Behnken design [46], two-level full factorial design (2^5^) [49,52,60], response surface methodology [52,60,72,81], Plackett–Burman design [64,81], one-factor experiment [64], L_18_-orthogonal array method [41] and central composite experimental design [49,67], are useful approaches for optimizing physico-chemical parameters for the production of fibrinolytic enzymes. For example, Farraj and colleagues (2020), applying a two-level full factorial design and response surface methodology, were able to increase the expression of the fibrinolytic enzyme isolated from *Bacillus flexus* using a solid state fermentation process. They demonstrated an enhanced production of fibrinolytic enzymes up to 3.5-fold [52].

## 6. Thrombolytic Activity of Marine Microbial Fibrinolytic Enzymes

The effective treatment of CVDs relies on thrombolysis agents, such as microbial fibrinolytic enzymes [82,83]. Microorganisms have been utilized to produce fibrinolytic enzymes since ancient times. In the last decades, researchers have intensively reported on the production of fibrinolytic enzymes from marine microorganisms. For example, a study carried out by Hwang et al. (2007) showed that BpKJ-31 is a promising candidate as a health-promoting biomaterial that does not induce bleeding [75]. Studies on in vitro lysis of clots by a purified fibrinolytic enzyme from the marine *Serratia marcescens* resulted in 38% clot lysis, which was significantly higher than that reported by streptokinase and heparin [62]. In addition, in the study carried by Gowthami and colleagues (2021), the fibrinolytic enzyme isolated from bacterial strain GPJ3 displayed digestion of blood clot completely under in vitro condition and exhibited potent activity on wound healing of macrophages [50]. The characteristics of the recombinant fibase from a marine *Bacillus subtilis* suggest its potential use for the treatment and/or prevention of thrombosis [71]. Moreover, purified PEKU1, a novel fibrinolytic protease from *Pseudomonas aeruginosa* KU1, has exceptional potential for being developed as a therapeutic agent to treat CVDs [47].

## 7. Conclusions

The scientific community already effectively utilizes all available information on fibrinolytic enzymes. As a future prospective, the community should focus on the exploration of novel sources of fibrinolytic enzymes, especially from the marine environment. Marine microbial fibrinolytic enzymes have immense therapeutic potential as target drugs to prevent or cure CVDs. Extensive studies on these enzymes promises to develop cost effective, safe and preventive solutions for the management of cardiac diseases. The new trend for developing and improving thrombolytic agents is to enhance its fibrin specificity and binding efficacy. Further optimization of production parameters is also required to design economical, effective and safe drugs. Thus, the use of marine microbial fibrinolytic enzymes as thrombolytic agents might be auspicious and a safe option in future.

## Figures and Tables

**Table 1:** Marine sources of fibrinolytic enzymes.

Isolated From	Microorganism	Enzyme	Reference
Marine sediment from Kovalam beach, Chennai, Tamil Nadu	*Streptomyces lusitanus*	-	[43]
Marine brown tube sponges *Agelas conifera*	*Streptomyces radiopugnans* VITSD8	-	[44]
Soil samples from South East Coast of India, Chennai	*Streptomyces rubiginosus* VITPSS1		[72]
Marine water sample	*Streptomyces venezuelae*	Thrombinase	[73]
Mangrove Sediments Pitchavaram, South East Coast of India	*Bacillus circulans*	-	[74]
Marine sediments of Ezhara beach, Kannur District, Kerala, India	*Pseudomonas aeruginosa* KU1	-	[46,47]
Mangrove sediments of Pulicat Lake, India	Bacterial strain GPJ3	-	[50]
South West Coast of India	*Bacillus flexus*	-	[52]
Mutagenesis of *B. subtilis* HQS-3	*Bacillus subtilis*	-	[53]
Surface seawater	*Bacillus vallismortis*	Bvsp	[55]
Fish scales, Kanyakumari, India	*Pseudoalteromonas* sp. IND11	-	[49]
Coast of Beihai prefecture of China	*Bacillus subtilis* HQS-3	-	[54]
Deep-sea sediment of Bay of Bengal	*Marinobacter aquaeolei* MS2-1	-	[51]
Jeotgal from gul (Oyster, *Crassostrea gigas*), korean fermented food	*Bacillus pumilus* BS15	AprEBS15	[29]
Marine niches covering 300 km of the western seacoast of Maharashtra, India	*Bacillus subtilis* ICTF-1	-	[41]
Oriyara beach in Kasargod district, Kerala, India	*Serratia rubidaea* KUAS001	-	[61]
Jeotgal from munggae (sea squirt), Korean fermented seafood	*Bacillus velezensis* BS2	AprEBS2	[59]
Sea mud	*Bacillus subtilis* WR350	-	[57]
Sea water collected from a depth of 10 m, 5 km away from Surathkal Coast in the Arabian Sea	*Serratia marcescens* subsp. *sakuensis* (KU296189.1)	-	[62,63,64,70]
Jeotgals from salted saeu (small shrimp), Korean fermented seafoods	*Bacillus subtilis* JS2	AprEJS2	[58]
Marine isolate	*Shewanella* sp. IND20	-	[60]
Jeotgal, Korean fermented seafood	*Bacillus licheniformis* KJ-31	BpKJ-31	[75]
Culture Collection of Algae, University of Texas, Austin	*Arthrospira* (*Spirulina*) *platensis*	-	[65]
University of Texas, Austin	*Chlorella vulgaris*	-	[66,67]
Dalian Institute of Chemical Physics, Chinese Academy of Sciences	*Tetraselmis subcordiformis*	-	[69]

**Table 2:** Purification strategies for isolating fibrinolytic enzymes from marine microorganisms.

Source	Enzyme	Purification Methods	Total Protein (mg)	Specific Activity (U mg^−1^)	Purification (Fold)	Yield (%)	References
*Bacillus flexus*	-	Ammonium sulphate precipitation (20%, 40% and 60%), Sephadex G-75 chromatography	4.4	315.2	5.2	10.8	[52]
*Bacillus pumilus* BS15	AprEBS15	Affinity chromatography by HiTrap IMAC FF column	-	-	-	-	[29]
*Bacillus velezensis* BS2	AprEBS2	Affinity chromatography by HiTrap IMAC FF column	-	131.15 m	-	-	[59]
*Bacillus subtilis* HQS-3	-	Ammonium sulphate precipitation, alkaline solution treatment, membrane concentration, dialysis, ion exchange and gel filtration chromatography	12	62,745	30	13	[54]
*Bacillus subtilis* JS2	AprEJS2	Affinity chromatography by HiTrap IMAC FF column	-	-	-	-	[58]
*Serratia marcescens* subsp. sakuensi	-	Ammonium sulfate precipitation (40%), dialysis, Fast protein liquid chromatograghy	0.03	1033	21.08	19.38	[62]
*Pseudomonas aeruginosa* KU1	-	Ammonium sulphate precipitation (50–80%), DEAE Sepharose, Sepharose 6B chromatography	0.8 mg∙mL^−1^	1491.50	13.52	17.79	[47]
*Bacillus licheniformis* KJ-31	BpKJ-31	DEAE-Sepharose FF column and gel filtration chromatography (HiPrep 16/60 Sephacryl S-200 HR column)	3.2	242.8	19	0.2	[75]
*Bacillus subtilis* ICTF-1	-	Ammonium sulfate precipitation (0–60%), UnoQ Sepharose Strong Anion Exchanger, Butyl Sepharose FF chromatography	0.669	280	32.42	7.5	[41]
*Streptomyces lusitanus*	-	Ammonium sulfate precipitation (60%), dialysis, size exclusion gel filtration chromatography	-	-	-	-	[43]
*Streptomyces radiopugnans* VITSD8	-	Ammonium sulphate precipitation (0–85%), dialysis, ion-exchange chromatography, Size exclusion chromatography	1.1	3891	22.36	35	[44]
*Arthrospira platensis*	-	Ammonium sulfate precipitation (40–70%), anion exchange (DEAE-Sephadex), size exclusion (Superdex 75) chromatography	0.02 mg∙mL^−1^	7988	32.72	28.85	[65]
*Chlorella vulgaris*	-	Acetone precipitation, anion exchange chromatography HiTrapTM DEAE FF cloumn	2.0	1834.6	2	4.0	[66]

**Table 3:** Some physicochemical characteristics of marine microbial fibrinolytic enzymes.

Source	Enzyme	Molecular Weight (kDa)	pH Opt.	Temp. Opt. (°C)	Activator/Co-Factor (Metal Ions)	Inhibitor	Class	References
*Bacillus flexus*	-	32	8	60	Mg^2+^, Mn^2+^	Zn^2+^, Fe^2+^ and Hg^2+^	-	[52]
*Bacillus pumilus* BS15	AprEBS15	27	8	40	K^+^, Mg^2+^, Zn^2+^	Na^+^, Fe^3+^, Mn^2+^, Co^2+^, PMSF, SDS, EDTA and EGTA	Serine protease	[29]
*Bacillus velezensis* BS2	AprEBS2	27	8	37	Mg^2+^, Ca^2+^, Mn^2+^	Fe^3+^, Zn^2+^, K^+^, Co^2+^, PMSF, EDTA, SDS	Serine protease	[59]
*Bacillus licheniformis* KJ-31	BpKJ-31	37	9	40	-	PMSF	Alkaline serine protease	[75]
*Bacillus subtilis* JS2	AprEJS2	24	8	40	K^+^, Mn^2+^, Mg^2+^, Zn^2+^	PMSF, EDTA, EGTA	Serine protease	[58]
*Bacillus subtilis* HQS-3	-	26	8	45–50	Mn^2+^,Ca^2+^, Mg^2+^	PMSF, EDTA, Cu^2+^, Zn^2+^ and Co^2+^	Serine metalloprotease	[54]
*Marinobacter aquaeolei* MS2-1	-	39	8	50	DTT	PMSF	Thiol-dependent serine protease	[51]
*Bacillus subtilis* ICTF-1	-	28	9	50	Ca^2+^	Zn^2+^, Fe^3+^, Hg^2+^ and PMSF	Serine protease	[41]
*Bacillus vallismortis*	Bvsp	34.4	6.5	54	Ca^2+^, Zn^2+^ and Ba^2+^	Na^+^, K^+^, NH4^+^ and Mg^2^^+^, PMSF, AEBSF, SDS, Guanidine-HCL, Urea and Isopropyl alcohol	Alkaline serine protease	[55]
*Serratia marcescens* subsp. sakuensi	-	43	7	55	Mn^2+^, Mg^2+^, Zn^2+^	PMSF, EDTA	Serine metalloprotease	[62]
*Pseudomonas aeruginosa* KU1	-	~50	-	-	Na^+^, K^+^ and Co^2+^	Fe^2+^, Mn^2+^ and Zn^2+^	Metalloprotease	[47]
*Shewanella* sp. IND20	-	55.5	8	50	Ca^2+^ and Mg^2+^	-	-	[60]
*Streptomyces lusitanus*	-	21	7	37	-	-	-	[43]
*Streptomyces radiopugnans* VITSD8	-	38	7	33	-	-	Serine endopeptidase	[44]
*Streptomyces rubiginosus* VITPSS1	-	45	-	-	-	-	-	[72]
*Arthrospira platensis*	-	72	6	40	Fe^2+^	PMSF	Serine metalloprotease	[65]
*Chlorella vulgaris*	-	45	-	-	Fe^2+^	PMSF, EDTA	Serine metalloprotease	[66]

**Table 4:** Fibrino(ogen) lytic activity of various marine microbial enzymes.

Source	Enzyme	Reference	Mode of Action	References
*Bacillus velezensis* BS2	AprEBS2	Strong α-fibrinogenase and moderate β-fibrinogenase	Direct	[59]
*Bacillus pumilus* BS15	AprEBS15	Strong α-fibrinogenase and moderate β-fibrinogenase activities	Direct	[29]
*Bacillus subtilis* JS2	AprEJS2	Strong α-fibrinogenase and moderate β -fibrinogenase activities	Direct	[58]
*Bacillus licheniformis* KJ-31	BpKJ-31	Strong Aα and fibrino (geno) lytic activity	Direct	[75]
*Bacillus subtilis* HQS-3	-	Hydrolyzed α chain of fibrin, followed by the β chain and finally the γ–γ chain	Direct	[54]
*Bacillus vallismortis*	Bvsp	Digest Aα- and Bβ-chains readily, but the γ-chain of fibrinogen slowly	Direct	[55]

**Table 5:** Kinetic properties of fibrinolytic enzymes.

Source	Enzyme	Substrate Specificity	V_max_	K_m_	k_cat_	k_cat_/K_m_	Reference
*Bacillus velezensis* BS2	AprEBS2	N-Succ-Ala-Ala-Pro-Phe-pNA	39.68 μM min^−1^	0.15 mM	18.14 s^−1^	1.25 × 10^5^ M^−1^s^−1^	[59]
*Bacillus pumilus* BS15	AprEBS15	N-succinyl-ala-ala-pro-phe- pNA	21.88 μM min^−1^	0.26 mM	10.02 s^−1^	3.83 × 10^4^ M^−1^s^−1^	[29]
*Bacillus subtilis* JS2	AprEJS2	N-Succ-Ala-Ala-Pro-Phe-pNA	16.71 μM min^−1^	0.09 mM	7.66 s^−1^	8.51 × 10^4^ M^−1^s^−1^	[58]
*Bacillus subtilis* ICTF-1	-	N-Succ-Ala-Ala-Pro-Phe-pNA	-	-	-	-	[41]
*Bacillus licheniformis* KJ-31	BpKJ-31	N-Succ-Ala-Ala-Pro-Phe-pNA	-	-	-	-	[75]
*Serratia marcescens* subsp. *sakuensis*	-	Fibrin	15.873 µmol min^−1^	0.66 mg mL^−1^	12.21 min^−1^	18.32 mL mg^−1^ min^−1^	[62]
*Bacillus subtilis*	Fibase	-	0.03 mM min^−1^	2.7 mmol L^−1^	-	-	[71]
*Bacillus vallismortis*	Bvsp	Fibrin	49.8 g mL^−^^1^ min^−^^1^	0.319 g mL^−^^1^	4.35 min^−^^1^	13.63 mL mg^−1^ min^−1^	[55]

**Table 6:** Cloning and expression parameters used for fibrinolytic enzymes production.

Bacterial Strain	Gene	Primer	Cloning Host	Cloning Vector	Expression Host	Expression Vector	References
*Bacillus velezensis* BS2	*aprEBS2*	CH51-F (5′-AGGATCCCAAGAGAGCGATTGCGGCTGTGTAC-3′, BamHI site underlined) CH51-R (5′-AGAATTCTTCAGAGGGAGCCACCCGTCGATCA-3′, EcoRI site underlined)	*B. subtilis* WB600	pHY300PLK	*E. coli* BL21 (DE3)	pETBS2	[59]
*Bacillus subtilis* JS2	*aprEJS2*	CH51-F (5′-AGGATCCCAAGAGAGCGATTGCGGCTGTGTAC-3′, BamHI site underlined) and CH51-R (5′-AGAATTCTTCAGAGGGAGCCACCCGTCGATCA-3′, EcoRI site underlined)	*B. subtilis* WB600	pHY300PLK	*E. coli* BL21 (DE3)	pHYJS2	[58]
*Bacillus pumilus* BS15	*aprEBS15*	CH51-F (5′-AGGATC CCAAGAGAGCGATTGCGGCTGTGTAC-3′, BamHI site underlined) and CH51-R (5′-AGAATTCTTCAGAGG GAGCCACCCGTCGATCA-3′, EcoRI site underlined)	*B. subtilis* WB600	pHY300PLK	*E. coli* BL21 (DE3)	pHYBS15	[29]
*Bacillus vallismortis*	*Bvsp*	BVSPF (5′-CGCGGATCC-ATGCAAGGTGAAATTAGGTTAATTCCATATTT-3′) containing BamH I and BVSPR (5′-CCGCTCGAGTCAGCCAATCTGTGCAAGTGGC-3′, Xho I sites (underlined)	-	-	*E. coli* BL21 (DE3)	pGEX-6P-bvsp	[55]
*Marinobacter aquaeolei* MS2-1	*-*	SPro F (5′-CCG GAT CCA TGG CGT TCA GCA AC-3′) and SPro R (5′-GGC TCG AGT TAG CGG GCA GGT GC-3′)	*E. coli*	pGEM-T	*E. coli* BL21 (DE3)	pET-28a-(+)	[51]
*Tetraselmis subcordiformis*	*rt-PA*	bar1F (5′-TCTGCACCATCGTCAACCACTACA-3′), bar1R (5′-TCAAATCTCGGTGACGGGCAGGAC-3′), rpa3F (5′-TCTTGGGCAGAACATACC-3′) and rpa3R (5′ -TCCCCCTGAACCTGAAAC-3′)	*-*	-	*E. coli* Top10	pSVrPA/CaMVbar	[69]
